# *Clerodendrum trichotomum* extract improves metabolic derangements in high fructose diet-fed rats

**DOI:** 10.1080/19768354.2021.2004221

**Published:** 2021-11-22

**Authors:** Mi Gyeong Jang, Jung Min Oh, Hee Chul Ko, Jae-Won Kim, Songyee Baek, Yeong Jun Jin, Sung-Pyo Hur, Se-Jae Kim

**Affiliations:** a Department of Biology, Jeju National University, Jeju, Republic of Korea; b Regional Innovation Center, Jeju National University, Jeju, Republic of Korea; c Jeju Institute of Korean Medicine, Jeju, Republic of Korea; d Korea Institute of Ocean Science & Technology, Busan, South Korea

**Keywords:** *Clerodendrum trichotomum*, dyslipidemia, insulin resistance, high fructose diet, metabolic disorders

## Abstract

*Clerodendrum trichotomum* has been reported to possess beneficial properties for human health, but its effects on metabolic syndrome have not been reported. In this study, we investigated the effect of *C. trichotomum* leaf extract (CT) on the metabolic derangements induced by a high-fructose (HF) diet. Sprague–Dawley rats were fed with a 46% carbohydrate diet (HC group), 60% high-fructose diet (HF group), or HF diet supplemented with CT (500 mg/kg of body weight/day, CT group) via drinking water for 16 weeks. Results showed that CT alleviated HF diet-induced insulin resistance, dyslipidemia, and hepatic steatosis In liver tissues, CT affected the signaling pathways of AMP-activated protein kinase, peroxisome proliferator-activated receptor α (PPARα), and sterol regulatory element binding protein 1. CT enriched the genes that were mainly involved in cytokine-cytokine receptor interaction, PPAR, PI3K-Akt signaling pathways, and fatty acid metabolism pathway. These results suggest that CT is a promising therapeutic against metabolic disorders.

## Introduction

Dietary habits characterized by a high consumption of fat and refined sugar promote metabolic derangements associated with symptoms of metabolic syndrome (Nakagawa et al. 2005; Tappy and Le 2010; Shin et al. 2020). High fructose (HF) intake has been associated with the development of metabolic syndrome symptoms, including hyperglycemia, hyperlipidemia, insulin resistance, and hepatic steatosis (Lim et al. 2010; Khitan and Kim 2013; Herman and Samuel 2016; Saklayen 2018; Feillet-Coudray et al. 2019). Unlike glucose, fructose does not elicit insulin secretion nor stimulate leptin secretion. However, it can supply an unregulated carbon skeleton for lipogenesis in the liver because of its metabolic characteristics (Basciano et al. 2005; Rutledge and Aldeli 2007; Douard and Ferraris 2013).

Metabolic stress, which is the extra- and intracellular buildup of metabolites derived from overnutrition, can activate inflammatory pathways and lead to chronic low-grade inflammation, which plays a key role in the initiation, propagation, and development of metabolic disorders (Baker et al. 2011). Treatments interfering with nuclear factor κB (NF-κB)-driven inflammation can alleviate type 2 diabetes and decrease hyperglycemia and insulin resistance in patients (Hotamisligil et al. 1993). Plant extracts that exhibit pleiotropic activities that can target multiple symptoms of metabolic disorders may have therapeutic potential for preventing or treating lifestyle diseases.

*Clerodendrum trichotomum* Thunb belongs to the family Verbenaceae and is widely distributed in East Asia, including Korea, Japan, Taiwan, and China. Its leaves, flowers, and twigs have been used as folk medicine for various inflammatory diseases, such as headaches and hypertension (Ann 2003). This plant contains various phytochemicals, such as sterols, flavonoids, glycosides, diterpenoids, and apigenin, that exhibit antioxidant and analgesic properties (Okigawa et al 1971; Kim et al. 2009; Wang et al. 2013). Its antimicrobial, antiviral, and hypouricemic effects have been reported *in vitro* and *in vivo* (Chathuranga et al. 2019; Jang et al. 2020). However, the preventive effects of *C. trichotomum* against metabolic syndrome have not yet been reported. In the present study, we investigated the effects of *C. trichotomum* leaf extract (CT) on metabolic derangements induced by a HF diet.

## Materials and methods

### Plant materials and extraction

*C. trichotomum* leaves were collected from the northern part of Jeju Island, Republic of Korea, in July 2018. The leaves were washed, dried at 60°C for 24 h, and then pulverized. The powder (100 g) was extracted for 24 h with 10 L of 70% ethanol at 25°C. The resulting ethanol extract (CT) was concentrated, freeze-dried, and stored at −70°C until use. High-performance liquid chromatography analysis confirmed that verbascoside (160.3 ± 2.4 mg/g) was the major component of CT (Supplementary Figure 1).

### Animals and experimental design

Four-week-old male Sprague–Dawley rats were purchased from Orient Bio (Seongnam, South Korea). The animals were acclimated for 3 weeks through housing in appropriate cages under a 12-h/12-h light/dark cycle at room temperature (23 ± 2°C) and 60 ± 5% relative humidity. The experiments were approved by the Institutional Animal Care and Use Committee of Jeju National University (No. 2019-0008). At the end of the acclimation period, the animals were randomly assigned to four groups (n = 6). The animals were fed with a 46% high-carbohydrate (HC) diet (HC group) instead of a normal diet to assess the difference in metabolic derangement caused by the HC and HF diets. The mice were fed with an HF diet without supplementation (HF group), with allopurinol (AP group), or with CT (CT group) for 16 weeks. For the CT group, CT (500 mg/kg of body weight) was administered to HF-fed rats via drinking water for 16 weeks. The compositions of the experimental diets are shown in Supplementary Table 1.

### Fasting blood glucose and oral glucose tolerance test (OGTT)

Fasting blood glucose levels were measured from blood samples drawn from the tail vein after fasting for 12 h at 2-week intervals during the 16-week experimental period. At the end of the final experimental day, an OGTT was performed. The experimental animals were fasted for at least 12 h, and then glucose (2 g/kg) was administered orally. After glucose administration, blood was collected from the tail vein at 30, 60, 90, and 120 min to measure blood glucose levels. Homeostasis model assessment of insulin resistance (HOMA-IR) indices were calculated by multiplying the fasting serum insulin and glucose, and then dividing the result by a constant number (Mathews et al. 1985).

### Biochemical parameter assays

Blood samples were drawn from the heart using a syringe and allowed to clot at 25°C. Blood serum was obtained using centrifugation at 3000 × g for 20 min at 4°C. The triglyceride (TG), total cholesterol (TC), high-density lipoprotein cholesterol (HDLC) and low-density lipoprotein cholesterol (LDL-C) levels were measured using commercial kits (DoGenBio, Seoul, South Korea). Glutamic oxaloacetic transaminase (GOT) and glutamic pyruvic transaminase (GPT) levels were measured using commercial kits from Asan Pharm (Gyeonggi, South Korea) according to the manufacturer’s protocol. Insulin levels were measured using rat insulin ELISA kits (Mercodia, Uppsala, Sweden).

### Histological analysis

Liver tissues were collected and fixed in 10% neutral-buffered formalin for 48 h. The tissues were dehydrated through an ethanol series, embedded in paraffin wax, and sectioned into 7 μm-thick slices. After deparaffinization and rehydration, sections were stained with hematoxylin and eosin (H&E). For immunohistochemical staining for tumor necrosis factor (TNF)-α, liver sections were deparaffinized in xylene, rehydrated in a graded alcohol series, and blocked with 0.3% H_2_O_2_ in water at 25°C. The tissue sections were washed with phosphate buffer solution and then incubated with a primary antibody against TNF-α (Abcam, Cambridge, UK). The sections were then incubated with horseradish peroxidase-conjugated secondary antibody (Vector Laboratories, Burlingame, CA, USA), washed, covered with 3,3′-diaminobenzidine, and counterstained with H&E. The stained sections were examined under a microscope (BX-51, Olympus, Tokyo, Japan).

### Western blot analysis

The liver tissue was homogenized in cold lysis buffer (1× RIPA buffer, 1 mM phenylmethylsulfonyl fluoride, 1 mM Na_3_VO_4_, 1 mM NaF, 1 μg/mL aprotinin, 1 μg/mL pepstatin, and 1 μg/mL leupeptin) and collected using centrifugation. The lysate protein concentrations were determined using a protein assay kit (Bio-Rad, Hercules, CA, USA). The proteins were separated using electrophoresis on sodium dodecyl sulfate–polyacrylamide gels and then transferred to polyvinylidene fluoride membranes. The membranes were blocked with 5% (w/v) bovine serum albumin and 0.1% (v/v) Tween 20 in Tris-buffered saline (TBST). After blocking, the membranes were incubated with fatty acid synthase (FAS), Sterol regulatory element-binding protein 1 (SREBP-1), peroxisome proliferator activated receptor alpha (PPARα), and β-actin antibodies (Santa Cruz, CA, USA) or AMP-activated protein kinase (AMPK), stearyl-CoA desaturase 1 (SCD-1), and p-AMPK antibodies (Cell Signaling Technology, Beverly, MA, USA) overnight at 4°C. The membranes were washed with 0.01% TBST and incubated at 25°C for 1 h with a peroxidase-conjugated secondary antibody. The membranes were washed with 0.01% TBST, and the proteins were detected using Westar ETA C 2.0 substrate (Cyanagen, Bologna, Italy).

### Transcriptome analysis

Total RNA was extracted from the liver tissues of rats in the HF and CT groups (*n* = 3) using QIAzol lysis reagent (Qiagen, Hilden, Germany) and then column-purified using the RNeasy Mini Kit (Qiagen). Libraries were independently prepared for each sample with 1 µg of total RNA using the Illumina TruSeq Stranded mRNA Sample Prep Kit (Illumina, San Diego, CA, USA). The indexed libraries were submitted for Illumina NovaSeq (Illumina) and paired-end (2 × 100 bp) sequencing was performed by Macrogen Inc. (Seoul, South Korea). Briefly, the raw reads from the sequencer were processed, and then the processed reads were aligned to the *Rattus norvegicus* (rn6) genome using HISAT v2.1.0 (Kim et al. 2015). The reference genome sequence of *Rattus norvegicus* (rn6) and annotation data were downloaded from the UCSC table browser (http://genome.uscs.edu). Transcript assembly and abundance estimation were performed using StringTie (Pertea et al. 2015; Pertea et al. 2016). The relative abundance of the gene was measured from read count values using StringTie. A statistical analysis to identify differentially expressed genes was performed using the estimates of abundance for each gene in the samples. To facilitate log2 transformation, one was added to each read count value for the filtered genes. The filtered data were log2-transformed and subjected to TMM normalization. The false discovery rate was controlled by adjusting the *p* value using the Benjamini–Hochberg algorithm. For the differentially expressed genes (DEGs) set, hierarchical clustering analysis was performed using complete linkage and Euclidean distance as a measure of similarity. Gene-enrichment, functional annotation, and pathway analyses for significant gene lists were performed using gProfiler (https://biit.cs.ut.ee/gprofiler/orth) and KEGG pathways (http://www.kegg.jp/kegg/).

### Statistical analysis

All statistical analyses were performed using SPSS version 21.0, for Windows (SPSS; Chicago, IL, USA). All data are presented as mean ± standard deviation (SD). Differences between groups were examined using one-way analysis of variance. Statistical significance was set at *p* < 0.05.

## Results

### CT supplementation decreased body weight gain

During the 16-week experimental period, the food and water intake of the rats were measured. As shown in Table 1, there were no significant differences in food and water intake among the HF and CT groups, but they differed in the HC group. There was no significant weight change in the HC and HF groups, but a significant change was observed in the CT group. While CT supplementation reduced the body weight gain in the HF diet-fed rats, it did not induce any toxicity.

**Table 1.** Effects of CT supplementation on body weight, food intake, and water intake in HF diet-fed rats.

Group	HC	HF	CT
Initial body weight (g)	322.5 ± 17.8^a^	322.9 ± 22.6^a^	310.3 ± 25.9^a^
Terminal body weight (g)	699.6 ± 70.0^a^	703.1 ± 42.5^a^	636.0 ± 42.8^b^
Body weight gain (g)	377.1 ± 57.1^a^	380.3 ± 28.2^a^	325.8 ± 24.4^b^
Food intake (g)	3778.4 ± 25.7^a^	4589.0 ± 41.2^b^	4546.0 ± 31.6^b^
Water intake (mL)	5197.3 ± 27.3^a^	6597.1 ± 37.5^b^	7016.3 ± 73.5^b^
Food efficiency ratio (%)	9.31 ± 1.29^a^	8.43 ± 0.49^a^	7.05 ± 0.45^b^

Data are presented as the mean ± SD (*n* = 6). Food efficiency ratio = body weight gain (g) for experimental period × 100/food intake (g) for the experimental period. Different letters indicate significant differences (*p* <0.05). SD, standard deviation; HC, 46% carbohydrate diet; HF, 60% fructose diet; CT, 60% fructose diet with *C. trichotomum* leaf extract (500 mg/kg of body weight).

### CT supplementation improves glucose homeostasis

**Figure 1.**
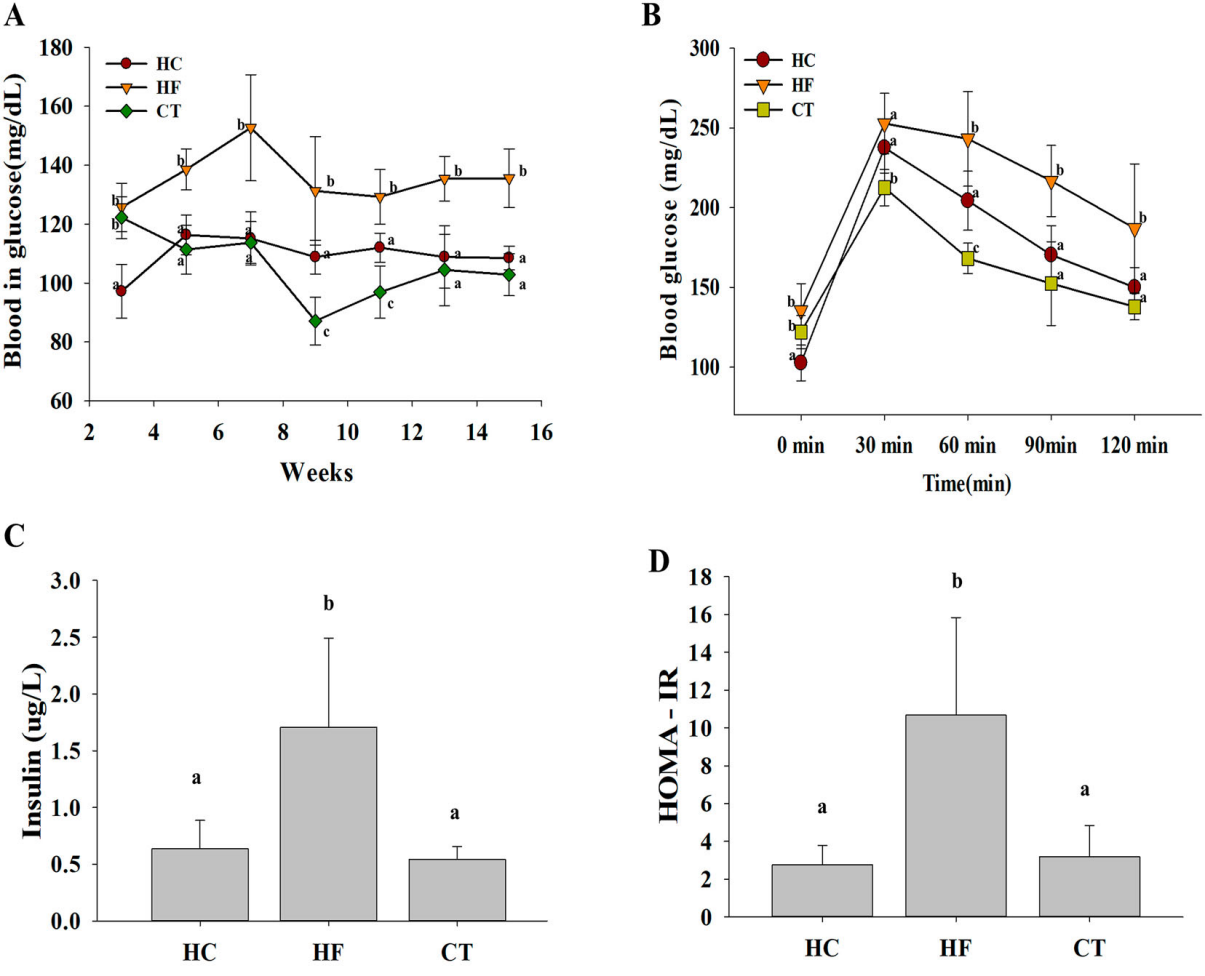
Effects of CT supplementation on glucose homeostasis in HF diet-fed rats over 16 weeks. (A) Fasting blood glucose profiles. (B) Oral glucose tolerance test. (C) Serum insulin levels. (D) HOMA-IR indices. Data are presented as means ± SDs (*n* = 6). Different letters indicate significant difference (*p* <0.05). HC, 46% carbohydrate diet; HF, 60% fructose diet; CT, 60% fructose diet with *C. trichotomum* leaf extract (500 mg/kg of body weight). HOMA-IR; homeostasis model assessment insulin resistance.

Fasting blood glucose level was higher in the HF group than in the HC group, indicating that the HF diet induced hyperglycemia in the animal model. As shown in Figure 1(A), CT supplementation attenuated HF diet-induced hyperglycemia. Meanwhile, the OGTT showed that the HF diet-induced oral glucose tolerance was alleviated by CT supplementation (Figure 1(B) and (C)). Additionally, CT supplementation effectively decreased the HOMA-IR in HF diet-fed rats (Figure 1(D)), suggesting that CT modulates glucose homeostasis.

### CT supplementation improves serum lipid profiles

**Figure 2.**
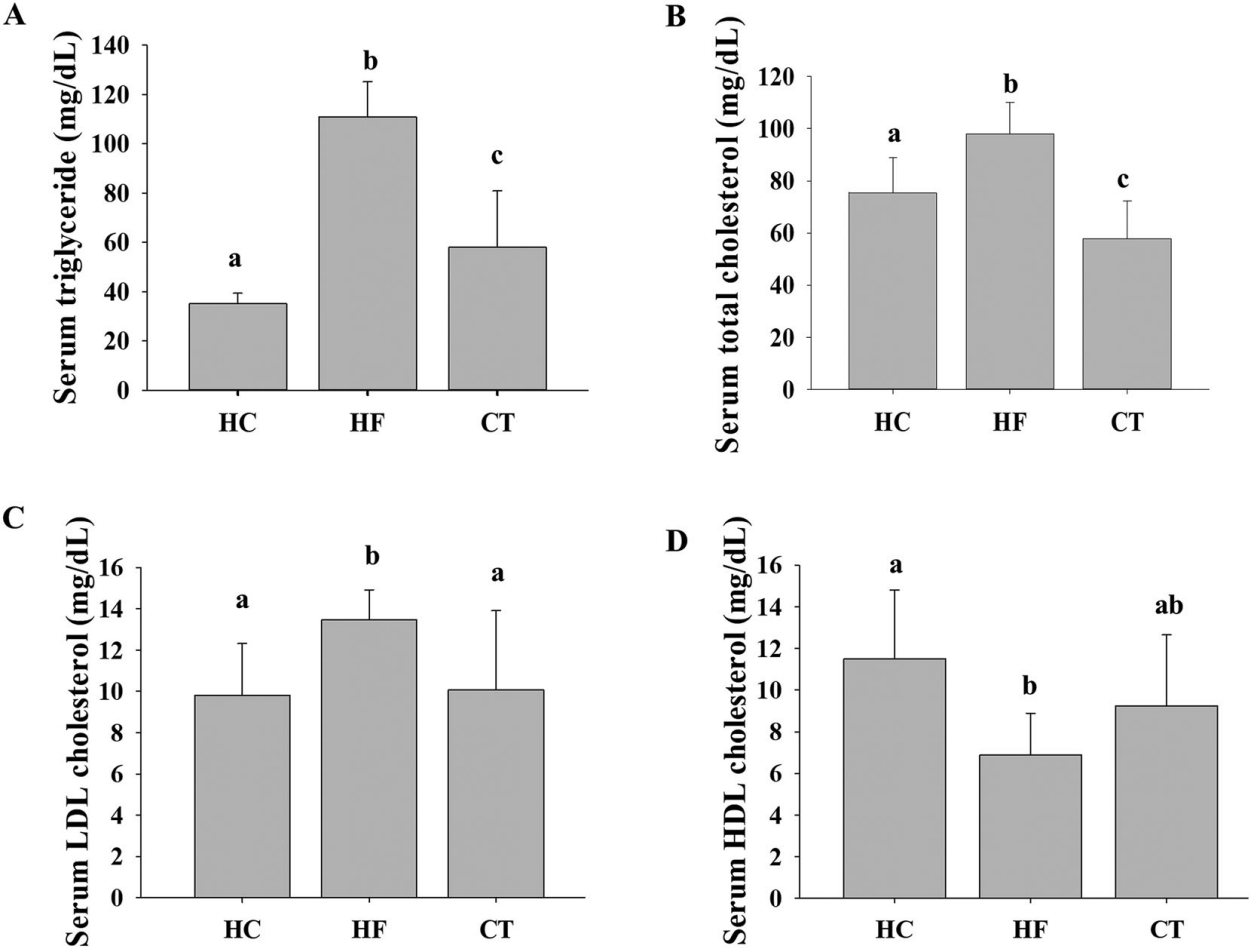
Effects of CT supplementation on serum lipid levels in HF diet-fed rats. (A) TG levels. (B) TC levels. (C) LDL-C levels. (D) HDL-C levels. Data are presented as means ± SDs. Different letters indicate significant difference (*p* <0.05). HC, 46% carbohydrate diet; HF, 60% fructose diet; CT, 60% fructose diet with *C. trichotomum* leaf extract (500 mg/kg of body weight). TG, triglyceride; TC, total cholesterol; LDL-C, low-density lipoprotein cholesterol; HDL-C, high-density lipoprotein cholesterol.

At the end of the 16-week experimental period, we compared the serum lipid profiles of all groups. Compared to the HC group, the HF group showed higher TG, TC, and LDL-C levels and a lower HDL-C level, indicating that the HF diet induced dyslipidemia in the animal model. CT supplementation effectively improved the derangement of serum lipid profiles in HF diet-fed rats (Figure 2).

### CT supplementation improves hepatic steatosis

**Figure 3.**
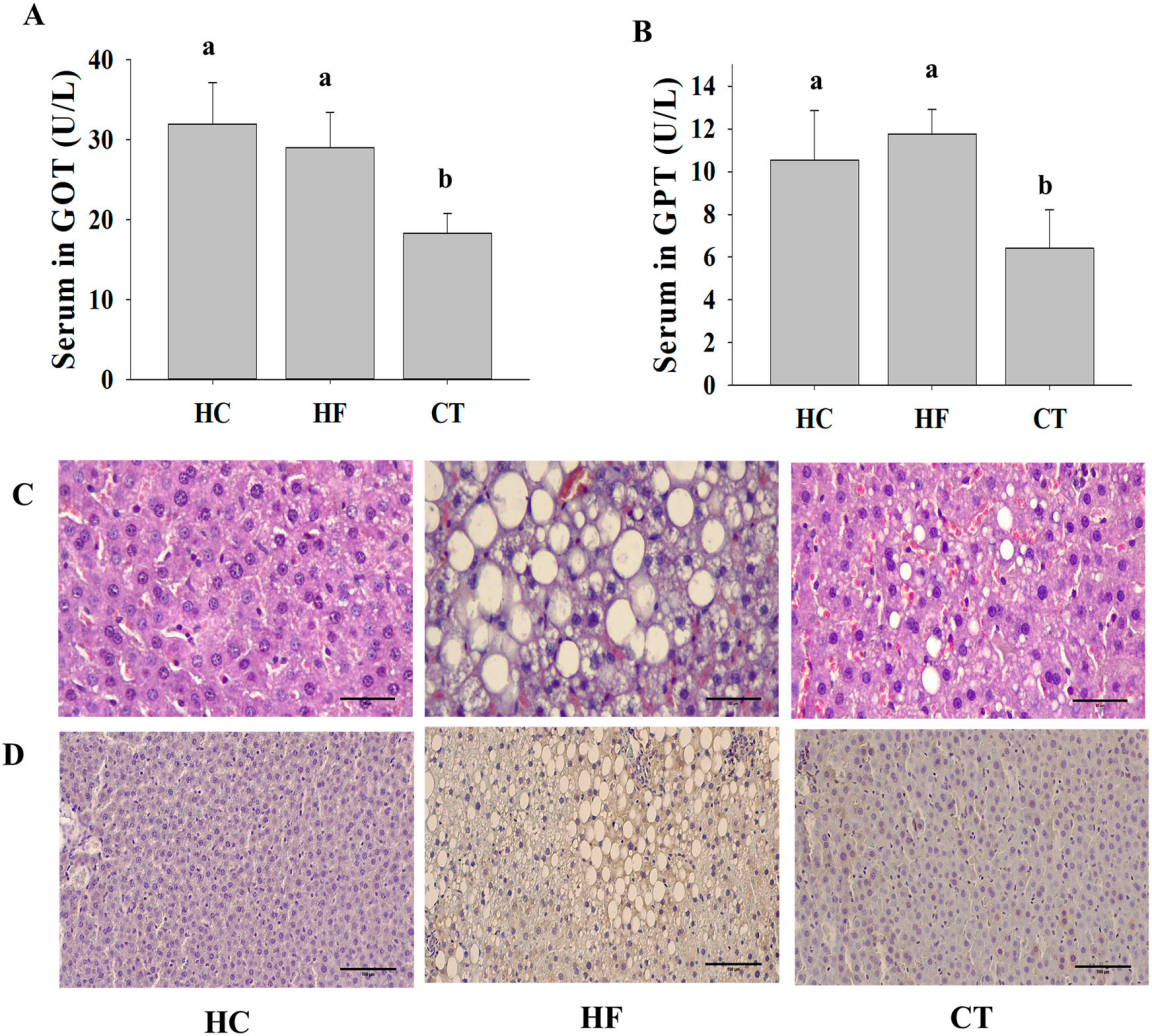
Effects of CT on liver function in HF diet-fed rats. (A) GOT levels. (B) GPT levels. (C) Hematoxylin and eosin (H&E) staining at 400 × magnification. Scale bar: 50 μm. (D) Immunohistochemical staining against TNF-α in liver paraffin sections at 200 × magnification. Scale bar: 100 μm. Data are presented as means ± SDs. Different letters indicate significant difference (*p* <0.05). HC, 46% carbohydrate diet; HF, 60% fructose diet; CT, 60% fructose diet with *C. trichotomum* leaf extract (500 mg/kg of body weight).

Serum GOT and GPT levels, which are sensitive clinical indicators of liver damage, were higher in the HF group than in the HC group. However, CT supplementation effectively reduced the GOT/GPT levels in HF diet-fed rats (Figure 3 (A) and (B)). Consistent with this result, histological analysis demonstrated that the HF group progressed to fatty liver by accumulating larger lipid droplets. However, CT supplementation ameliorated HF diet-induced hepatic steatosis (Figure 3(C)). In addition, immunohistochemistry of TNF-α in liver tissues revealed that CT supplementation markedly reduced HF diet-induced TNF-α expression (Figure 3(D)).

### CT supplementation modulates the lipid metabolism in the liver tissue

**Figure 4.**
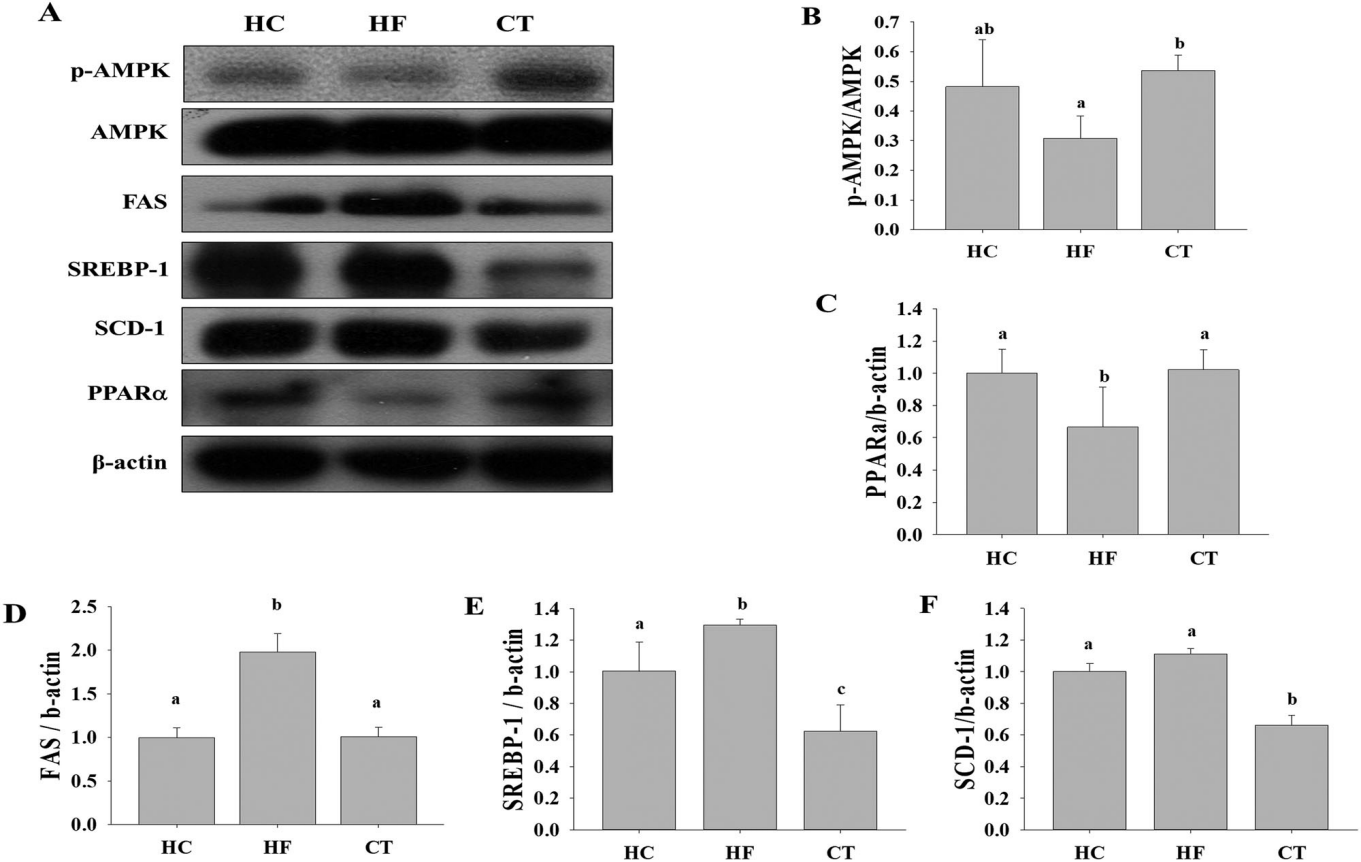
(A) Western blot analysis for proteins associated with lipid metabolism among groups. Relative expression levels of (B) AMPK, (C) PPARα, (D) SREBP-1, (E) FAS, and (F) SCD-1 were determined using densitometry. Results are expressed as the means ± SDs (*n* = 3). Different letters indicate significant difference (*p* <0.05). HC, 46% carbohydrate diet; HF, 60% fructose diet; CT, 60% fructose diet with *C. trichotomum* leaf extract (500 mg/kg of body weight).

To explore the underlying molecular mechanisms of the beneficial effects of CT supplementation, we investigated the expression of hepatic proteins among the groups. SREBP-1, (SCD-1), and (FAS), which are associated with *de novo* lipogenesis, were significantly higher in the HF group than in the HC group (Figure 4). However, CT supplementation significantly decreased the expression of SREBP-1, SCD, and FAS. CT supplementation increased AMPK phosphorylation and PPARα expression. These results suggest that CT exerts beneficial effects by modulating lipid metabolism in HF diet-fed rats.

### CT supplementation modulates the liver transcriptome profiles

**Figure 5.**
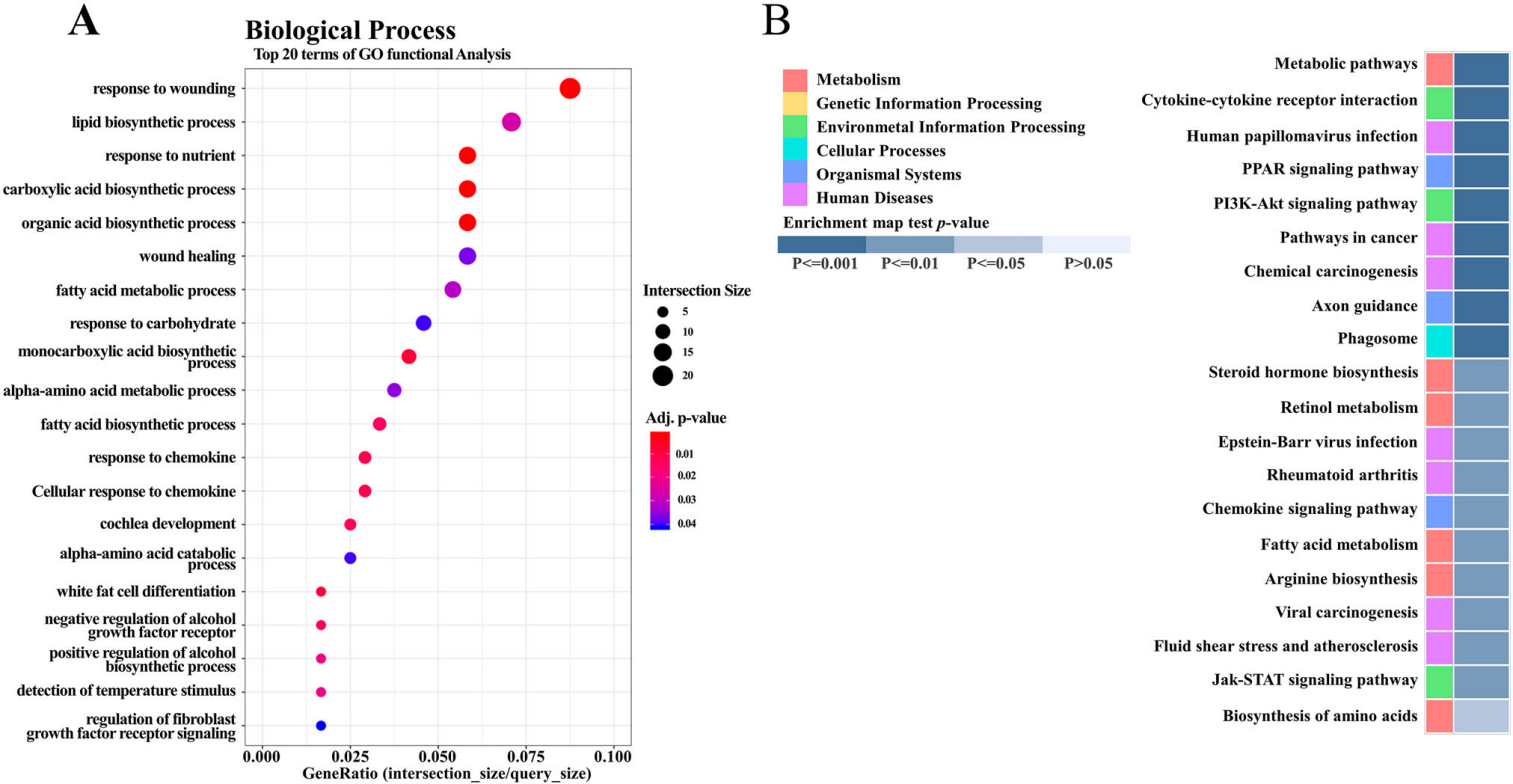
Effects of CT supplementation on liver transcriptome profiles in HF diet-fed rats. (A) Gene ontology (GO) analysis. Top 20 terms of GO function analysis in biological process are shown. (B) KEGG pathway analysis. Top 20 terms of KEGG pathway are shown. *p* value according to modified Fisher’s exact test.

To explore how CT supplementation affected overall gene expression in HF diet-fed rats, we further analyzed the liver transcriptome profiles of the HF and CT groups (*n* = 3). We identified a total of 392 DEGs (*p* < 0.05, fold change > 2), which comprised 199 upregulated genes and 193 downregulated genes in CT versus HF (Supplementary Figure 2(A)). Hierarchical clustering analysis showed similar gene expression patterns among individuals within the HF or CT groups, but not between the groups (Supplementary Figure 2(B)). To understand the significance of these DEGs, we performed gene ontology (GO) and Kyoto Encyclopedia of Genes and Genomes (KEGG) pathway analyses. The top GO terms in biological processes were related to the response to wounding, lipid biosynthesis, fatty acid metabolic process, and response to carbohydrates (Figure 5(A)). The KEGG pathway analysis showed that CT supplementation mainly affected the metabolic pathway, cytokine–cytokine receptor interaction, PPAR signaling pathway, PI3K–Akt signaling pathway, and fatty acid metabolism pathway (Figure 5(B)).

## Discussion

In this study, we investigated the preventive effects of CT on HF diet-induced metabolic derangement using an HC diet containing corn starch instead of a normal diet. The dose concentration (500 mg/kg of body weight) of CT in animals was determined based on previous reports (Choo et al. 2015; Jang et al. 2020). Consequently, the present study demonstrated that CT supplementation effectively improved HF diet-induced oral glucose tolerance, insulin resistance, dyslipidemia, and hepatic steatosis.

These beneficial effects of CT supplementation might be mediated by the anti-inflammatory activities of CT and its major constituent, verbascoside (Korkina 2007; Alipieva et al. 2014). Inflammatory cytokines promote the progression of fatty liver to fatty hepatitis, and serum and liver TNF-α levels have been reported to increase in patients with nonalcoholic fatty liver disease (Haukeland et al. 2006; Petta et al. 2009; Jarukamjorn et al. 2016). Inhibition of hepatic NF-κB signaling efficiently prevents liver steatosis and inflammation when rodents are fed an HF diet (Wunderlich et al. 2008). Tissues with accumulated fat release numerous inflammatory cytokines, including TNF-α, IL-6, and COX-2, which can activate cascades of inflammatory signaling (Kershaw and Flier 2004). In the present study, CT supplementation decreased the expression of TNF-α protein, but it increased the expression of of PPARα in liver tissues. PPARα plays a crucial role in fatty acid catabolism and clearance, as well as sphingolipid metabolism in the liver. It also has anti-inflammatory properties by counteracting NF-κB and enhancing fibroblast growth factor 21 (Li et al. 2019; Wang et al. 2020).

AMPK plays a major role in maintaining energy balance by controlling lipid metabolism in the liver (Peng et al. 2019). The dysfunction of AMPK signaling is strongly correlated with insulin resistance, obesity, and dyslipidemia, as well as metabolic disorders associated with diabetes (Luo et al. 2005; Ruderman et al. 2013). AMPK promotes fatty acid oxidation by mediating the expression of the fatty acid oxidation-related genes, carnitine palmitoyl transferase 1a (CPT1A), and PPARα (Kohjima et al. 2008). The present study showed that CT supplementation activated AMPK but decreased SREBP-1, SCD-1, and FAS expression in HF diet-fed rats. Consistent with these results, HF feeding in animals reportedly affected the expression of genes involved in the PPAR signaling pathway, as well as fatty acid and glucose metabolism (Li et al. 2016). In addition, fructose consumption decreased PPARα expression and caused the transcriptional inhibition of CPT1A, resulting in liver lipid accumulation (Ohashi et al. 2015), and SCD-1-gene-deficient mice had less liver fat accumulation (Roberts et al. 2015).

The liver transcriptome analysis showed that CT supplementation mainly enriched GO terms associated with biological processes, such as lipid biosynthesis and fatty acid metabolic processes, and response of carbohydrates and chemokines. Similarly, the KEGG pathway analysis showed that CT regulated genes involved in the PPAR signaling pathway, cytokine–cytokine receptor interaction, fatty acid metabolism pathway, PI3K–Akt signaling pathway, and metabolic pathways. The changes in transcriptome profiles after CT supplementation were consistent with the results from the western blot analysis. Since CT supplementation affected the KEGG pathways that are closely related to HF diet-induced metabolic derangements, we hypothesized that CT might alleviate HF diet-induced metabolic derangements by regulating the genes relevant to metabolic dysfunction in HF diet-fed rats. Thus, *C. trichotomum* leaf might be a potential therapeutic against lifestyle-related metabolic disorders.
